# Two New Oleanane-Type Triterpenes Isolated from Japanese Post-Fermented Tea Produced by Anaerobic Microbial Fermentation

**DOI:** 10.3390/molecules18054868

**Published:** 2013-04-24

**Authors:** Yong-Lin Huang, Sachi Nagai, Takashi Tanaka, Yosuke Matsuo, Yoshinori Saito, Isao Kouno

**Affiliations:** 1 Department of Natural Product Chemistry, Graduate School of Biomedical Sciences, Nagasaki University, 1-14 Bunkyo-machi, Nagasaki 852-8521, Japan; E-Mails: hylgxib@live.cn (Y.-L.H.); bb55612025@cc.nagasaki-u.ac.jp (S.N.); y-matsuo@nagasaki-u.ac.jp (Y.M.); saiyoshi@nagasaki-u.ac.jp (Y.S.); ikouno@nagasaki-u.ac.jp (I.K.); 2 Guangxi Key Laboratory of Functional Phytochemicals Research and Utilization, Guangxi Institute of Botany, Guilin 541006, China

**Keywords:** post-fermented tea, anaerobic fermentation, triterpene, oleanane, Awa-bancha

## Abstract

Four triterpenes were isolated from a traditional Japanese tea product produced by anaerobic microbial fermentation of heated green tea leaves (*Camellia sinensis*). Two of the compounds were novel and characterized by spectroscopic investigation to be 13,26-epoxy-3β,11α-dihydroxyolean-12-one and 3β,11α,13β-trihydroxyolean-12-one. Two known triterpenes were identified as taraxastane-3β,20β-diol and taraxastane-3β,20α-diol. These triterpenes were not detected in the original green tea leaves.

## 1. Introduction

Green tea is the most common tea beverage in Japan and China. Many biological, nutritional, and epidemiological studies have confirmed the health benefits of green tea, including antioxidative activity and prevention of cardiovascular diseases and cancer [1]. Green tea is produced from fresh leaves of *Camellia sinensis* (L.) O. Kuntze (Theaceae) by steaming or panning just after harvest, followed by systematic processes of kneading, crumpling, and drying. The first heating treatment inactivates enzymes that catalyze the degradation of the chemical constituents. Thus, the polyphenol composition of green tea is similar to that of fresh leaves. In some parts of East Asia, the heated green tea leaves are further fermented by microorganisms to produce post-fermented tea [2]. In Southern China, the tea produced by aerobic fermentation is called ripe pu-erh tea or dark tea, which contains caffeine and uncharacterized catechin degradation products [3,4]. Recently, some characteristic catechin oxidation products were isolated from a tea treated with *Aspergillus* spp. under aerobic conditions [5,6]. Conversely, some teas in Myanmar, Southern China, and the Shikoku island of Japan are produced by anaerobic microbial fermentation [2]. Our previous study revealed that the typical products from the Shikoku region contained catechin reduction products that were identical to the metabolites of tea catechin produced by mammalian intestinal bacteria [7,8,9]. In subsequent chemical investigations of the characteristic post-fermented tea, we isolated four triterpenes including two new compounds from a typical tea produced by anaerobic fermentation. This paper deals with the isolation and structure elucidation of these compounds.

## 2. Results and Discussion

**Figure 1:**
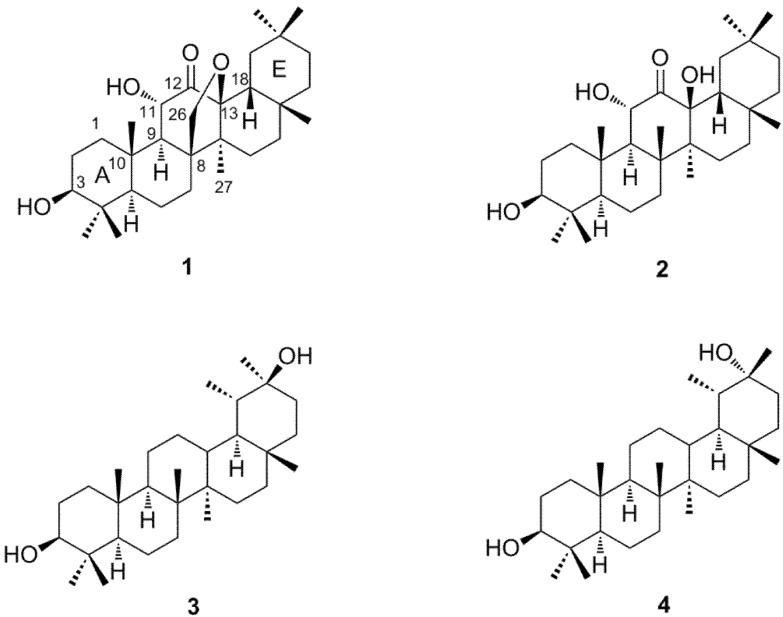
Structures of compounds **1**–**4**.

Fractionation of the aqueous acetone extract of the post-fermented tea by Diaion HP20SS column chromatography, followed by TLC analysis of the non-polar fraction showed the presence of terpenoids that were not detected in the corresponding fraction of green tea leaves. Separation of the non-polar fraction by silica gel and Chromatorex ODS column chromatography afforded four triterpenes (**1**–**4**, Figure 1). Compounds **1** and **2** were found to be new compounds. Compounds **3** and **4** were known compounds and identified as taraxastane-3β,20β-diol [10] and taraxastane-3β,20α-diol [11,12] by comparison of their spectroscopic data with literature values. 

Compound **1 **was obtained as an off-white amorphous powder, and its molecular formula C_30_H_48_O_4_ was determined by HR-ESI-MS analysis, which showed the [M+Na]^+^ peak at *m/z* 495.3438 (calculated for C_30_H_48_NaO_4_, 495.3445). The IR spectrum of **1** showed absorptions arising from hydroxyl (3,462 cm^−1^) and carbonyl (1,716 cm^−1^) functional groups. The ^13^C-NMR spectrum of **1** showed 30 carbon signals including four oxygen bearing carbons [δ 71.8, 71.9, 78.4, and 89.2] and a carbonyl carbon [δ 208.4]. The ^1^H-NMR spectrum showed signals corresponding to seven tertiary methyl groups at δ 0.77, 0.85, 0.87, 0.89, 0.99, 1.00, and 1.09. The spectrum also indicated the presence of an oxygen bearing methylene group [δ 3.75 (1H, dd, *J* = 9.2, 1.3 Hz) and 4.27 (1H, d, *J* = 9.2 Hz)] and two oxymethine protons [δ 3.21 (1H, dd, *J* = 4.8, 11.6 Hz) and 4.30 (1H, d, *J* = 10.4 Hz)]. These NMR data and the unsaturation index (UI = 7) derived from the molecular formula suggested that **1** is a pentacyclic triterpene with a ketone group and an ether ring. Assignment of the signals with the aid of HSQC and HMBC spectroscopy showed that the signals arising from the A and B ring carbons were similar to those of compounds **3** and **4 **(Table 1). 

**Table 1:** ^1^H and ^13^C-NMR data for **1** and **2** (500 MHz, in CDCl_3_).

position	1		2	
	δC	δH	δC	δH
1	39.3	1.20 dd (15.2, 5.6)	40.0	1.01 m
		2.24 m		2.55 m
2	27.2	1.55 m	27.5	1.54 m
		1.62 m		1.59 m
3	78.4	3.21 dd (11.6, 4.8)	78.5	3.18 dd (10.3, 5.9)
4	38.9		39.4	
5	54.7	0.75 dd (11.9, 1.9)	55.3	0.70 dd (11.1, 3.3)
6	19.5	1.09 m	17.7	1.51 m
		1.68 m		1.58 m
7	29.1	1.27 ddd (13.4, 12.8, 4.4)	34.0	1.30 m
		1.77 ddd (13.4, 3.0, 2.5)		1.43 m
8	50.9		43.3	
9	54.8	1.41 dd (10.4, 1.3)	56.9	1.48 d (12.1)
10	38.9		39.5	
11	71.8	4.30 d (10.4)	72.4	4.89 d (12.1)
12	208.4		212.7	
13	89.2		82.3	
14	46.9		45.2	
15	27.8	1.12 m	22.6	1.06 m
		1.70 m		2.09 ddd (13.5, 13.2, 4.1)
16	25.8	0.85 m	30.5	1.17 m
		1.98 ddd (13.9, 13.7, 4.1)		1.82 ddd (13.6, 13.4, 4.1)
17	32.7		33.6	
18	39.7	2.02 dd (13.5, 2.5)	49.0	1.50 dd (13.3, 1.8)
19	35.6	1.09 t (13.5)	38.5	1.25 t (13.3)
		2.24 dd (13.5, 2.5)		1.97 dd (13.3, 1.8)
20	30.9		31.4	
21	34.5	1.12 m	34.0	1.16 m (2H)
		1.31 ddd (13.9, 13.4, 4.1)		
22	38.6	1.13 m	39.0	1.21 m
		1.59 m		1.37 m
23	28.4	0.99 s (3H)	28.2	0.99 s (3H)
24	15.7	0.77 s (3H)	15.3	0.81 s (3H)
25	15.8	1.09 s (3H)	16.0	1.11 s (3H)
26	71.9	3.75 dd (9.2, 1.3)	20.5	1.36 s (3H)
		4.27 d (9.2)		
27	16.5	0.85 s (3H)	18.5	0.91 s (3H)
28	29.4	1.00 s (3H)	31.3	1.22 s (3H)
29	33.4	0.87 s (3H)	31.9	0.89 s (3H)
30	23.4	0.89 s (3H)	25.0	0.96 s (3H)

**Figure 2:**
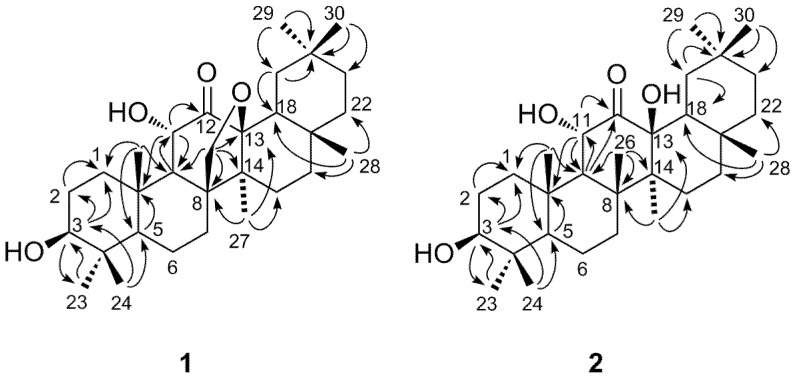
Key HMBC correlations (H→C) of **1** and **2**.

**Figure 3:**
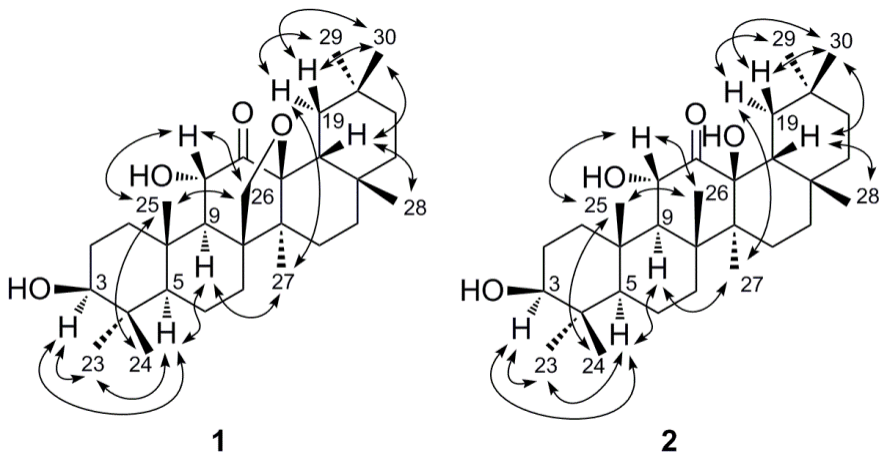
Key NOE correlations of **1** and **2**.

HMBC correlations (Figure 2) of an oxymethine proton at δ 4.30 (1H, d, 10.4 Hz) with the C-9 (δ 54.8) and C-10 (δ 38.9) assigned this proton signal to H-11, and the correlation of the H-11 with the carbonyl carbon signal at δ 208.4 revealed the C-12 keto structure of **1**. The oxygen bearing methylene proton signals at δ 3.75 and 4.27 were assigned to H_2_-26, because these protons were correlated with C-8 (δ 50.9), C-9 (δ 54.8) and C-14 (δ 46.9). The C-8 and C-14 were also correlated with a methyl proton signal at δ 0.85, which was assigned to H_3_-27. The H_3_-27 and H_2_-26 showed cross peaks with an oxygen-bearing quaternary carbon at δ 89.2. These correlations unequivocally indicated the ether linkage between C-26 and C-13. The assignments of the remaining proton and carbon signals of D and E rings were achieved by the HSQC and HMBC correlations illustrated in Figure 2, confirming the pentacyclic structure. The large vicinal coupling constants of H-3 and H-11 (Table 1) indicated the equatorial orientation of the hydroxyl groups of these carbons. In addition, the NOESY correlations illustrated in Figure 3 were in agreement with usual oleanane-type structures. Based on these spectroscopic results, the structure of compound **1** was determined to be 13,26-epoxy-3β,11α-dihydroxyolean-12-one.

The molecular formula of **2** was determined to be C_30_H_50_O_4_ by HR-ESI-MS ([M+Na]^+^ at *m/z* 497.3583, calculated for C_30_H_50_NaO_4_, 497.3601). The molecular weight was 2 mass units larger than that of **1**, and the UI (6) of **2** was smaller than that of **1** (UI = 7). The IR spectrum of **2** showed absorptions of hydroxyl (3,402 cm^−1^) and carbonyl (1,707 cm^−1^) groups. The ^1^H and ^13^C-NMR spectra were closely related to those of **1**, except for the appearance of eight methyl singlets and the disappearance of the oxymethylene group (C-26) of **1 **(Table 1). ^1^H-^1^H COSY, HSQC, and HMBC spectroscopy confirmed the plane structure, which had a C-26 methyl group (δ 20.5) instead of the C-26 oxymethylene (δ 71.9) of **1** (Figure 2). The large vicinal coupling constants of H-3 (*J*_2__ax,3_ = 10.3 Hz) and H-11 (*J*_9,11_ = 12.1 Hz) indicated the equatorial configuration of the hydroxyl groups at these positions. In addition, the NOESY correlations shown in Figure 3 indicated that the stereostructure of **2** was the same as that of **1**. Based on these spectral data the structure of **2** was determined to be 3β,11α, 13β-trihydroxyolean-12-one.

The origin of the four triterpenes isolated in this experiment is ambiguous at present because **1**–**4** were not detected in the non-polar fraction of green tea leaves by TLC comparison. Although the tea plant contains saponins, the triterpenes found are different from the aglycones of tea leaf saponins, which are oleanane-type triterpenes with a different substitution pattern of oxygen functional groups [13,14]. However, 3-*O*-acetate of **2** had been isolated from *Gordonia ceylanica* (Theaceae) [15]. The known compound **4** was identified as a component of the nonsaponifiable lipid of the seed oil of *Camellia japonica* L., which belongs the same genus as the tea plant [16]. Therefore, 12-keto-13β-oxyoleanane triterpenes may be originally present in the green tea. More recently, 7-keto-13β-oxyoleanane triterpenes were isolated from Chinese post-fermented tea [17]; therefore, a more detailed study of the triterpenes of tea including possible participation of bacteria in molecular conversion of triterpenes may be necessary.

## 3. Experimental

### 3.1. Materials

Post-fermented tea, Awa-bancha, was purchased in September 2010 at a local market in Tokushima City, Japan.

### 3.2. Analytical Procedures

IR spectra were obtained with a JASCO FT/IR-410 spectrophotometer, and optical rotations were measured with a JASCO DIP-370 digital polarimeter (Jasco Co., Tokyo, Japan). ^1^H and ^13^C-NMR spectra were measured in CDCl_3_ at 27 °C, using a Varian Unity plus 500 spectrometer (500 MHz for ^1^H and 125 MHz for ^13^C) (Varian, Palo Alto, CA, USA). Coupling constants are expressed in Hz and chemical shifts are given on a *δ* (ppm) scale. High resolution-electrospray ionization mass spectra (HR-ESI-MS) were obtained using a JEOL JMS-T100TD spectrometer (JEOL Ltd., Tokyo, Japan).

Column chromatography was performed using a Diaion HP20SS (Mitsubishi Chemical, Tokyo, Japan). Silica gel (100–210 μm, Kanto Chemical Co., Tokyo, Japan) and Chromatorex ODS (100–200 mesh, Fuji Silysia Chemical Ltd., Kasugai, Japan) were used for column chromatography. Thin-layer chromatography was performed on precoated silica gel 60 F_254_ (0.2 mm thick, Merck KGaA, Darmstadt, Germany) with CHCl_3_/MeOH/H_2_O (9:1:0.1 or 8:2:0.2, v/v/v) or acetone-hexane (1:1 or 2:1, v/v) as the mobile phase. Spots were detected using 5% sulfuric acid reagent followed by heating.

### 3.3. Extraction and Separation

Post-fermented tea (Awa-bancha, 1.5 kg) was powdered by a Waring blender and extracted with acetone/H_2_O (6:4, v/v, 10 L) by maceration at room temperature. The extraction was repeated three times. After filtration, the extract was concentrated under reduced pressure to remove the organic solvent, and the resulting precipitates were dissolved by the addition of MeOH (30% of the total volume). The solution was applied to a Diaion HP20SS column (8 × 15 cm) with 30% MeOH. Increasing proportions of MeOH (10% stepwise gradient, each 1 L) were added to give seven fractions: fr. 1 (343 g), fr. 2 (64 g), fr. 3(47 g), fr. 4 (29 g), fr. 5 (57 g), fr. 6 (17 g) and fr. 7 (22 g). Fraction 1 mainly contains sugars and lactic acid, and fractions 2–6 contain catechins and related polyphenols [8]. Fraction 7 was applied to a silica gel column (5 × 25 cm) with CHCl_3_/MeOH as the mobile phase (10:0 → 0:10, v/v, 10% stepwise, each 300 mL) to give seven fractions. Fraction 7–1 (9.5 g) was further purified by silica gel column chromatography (3 × 25 cm) with hexane-acetone as the mobile phase (0:1 → 5:5, v/v), obtaining seven fractions. Fraction 7-1-2 (1.3 g) was purified using a Chromatorex ODS column (3 × 25 cm) with 80%–100% MeOH in H_2_O as the mobile phase (5% stepwise, each 200 mL) to yield compound **1** (47 mg), and four sub-fractions: fr. 7-1-2-2 (510 mg), fr. 7-1-2-3 (43 mg), fr. 7-1-2-4 (70 mg) and fr. 7-1-2-5 (176 mg). Fraction 7-1-2-2 was purified by silica gel chromatography (3 × 20 cm) with 1%–5% acetone in CHCl_3_ as the mobile phase (1% stepwise, each 100 mL) to yield compound **2** (38 mg). Fraction 7-1-2-5 was subjected to silica gel chromatography (2 × 20 cm) with 1%–5% acetone in CHCl_3_ as the mobile phase (1% stepwise, each 100 mL) to yield compounds **3** (18 mg) and **4** (2.2 mg).

### 3.4. Physicochemical Data of Compounds **1** and **2**

*13,26-Epoxy-3β,11α-dihydroxyolean-12-one* (**1**): white amorphous powder; [α]_D_^32^ +35.6 (*c* = 0.08, CHCl_3_); ESI-MS *m/z*: 495 [M+Na]^+^; HR-ESI-MS *m/z*: 495.3438 (calculated for C_30_H_48_NaO_4_, 495.3445); IR ν_max_ cm^−1^: 3,462, 2,939, 1,716, and 1,458. See Table 1 for ^1^H and ^13^C-NMR.

*3β,11α,13β-Trihydroxyolean-12-one* (**2**): yellow amorphous powder; [α]_D_^32^ +1.4 (*c* = 0.09, CHCl_3_); ESI-MS *m/z*: 497 [M+Na]^+^; HR-ESI-MS *m/z*: 497.3583 (calculated for C_30_H_50_NaO_4_, 497.3601); IR ν_max_ cm^−1^: 3,402, 2,949, 1,707, and 1,462. See Table 1 for ^1^H and ^13^C-NMR.

## 4. Conclusions

Four triterpenes that were not detected in the original green tea leaves were isolated from a post-fermented tea produced by anaerobic microbial fermentation of green tea leaves. Two of the compounds were novel and characterized by spectroscopic investigation to be 13,26-epoxy-3β,11α-dihydroxyolean-12-one and 3β,11α,13β-trihydroxyolean-12-one. To our knowledge, compound **1** is the first oleanane-type triterpene with an ether linkage between C-13 and C-26. The presence of a ketone group at C-12 is a characteristic feature of **1** and **2**, and the biogenetical relationship between **1** and **2** are also interesting; that is, it remains to be determined whether the reductive cleavage of the ether linkage between C-13 and C-26 of **1** produced **2** or **1** was generated by the dehydrogenation of **2**. A more detailed study of the triterpenes of fermented tea products including possible participation of bacteria in molecular conversion of triterpenes may be necessary.
